# Efficacy of galcanezumab in patients with migraine and history of failure to 3–4 preventive medication categories: subgroup analysis from CONQUER study

**DOI:** 10.1186/s10194-021-01322-7

**Published:** 2021-09-30

**Authors:** Rose Okonkwo, Antje Tockhorn-Heidenreich, Chad Stroud, Marie-Ange Paget, Manjit S. Matharu, Cristina Tassorelli

**Affiliations:** 1 Eli Lilly and Company, Medical Affairs CRS, Indianapolis, IN 46285 USA; 2 University College London (UCL) Queen Square Institute of Neurology and National Hospital for Neurology and Neurosurgery, London, UK; 3 Headache Science and Neurorehabilitation Center, IRCCS Mondino Foundation, Pavia, Italy; 4 Department of Brain and Behavioral Sciences, University of Pavia, Pavia, Italy

**Keywords:** Difficult-to-treat, Prior preventive, Calcitonin gene-related peptide, CGRP, Migraine headache days

## Abstract

**Background:**

Chronic migraine (CM) and episodic migraine (EM) are associated with substantial headache-related disability, poor quality of life and global societal burden. In this subgroup analysis from the CONQUER study, we report efficacy outcomes from a pre-specified analysis of galcanezumab versus placebo in patients with CM or EM and 3–4 prior preventive medication category failures due to inadequate efficacy (after at least 2 months at maximum tolerated dose), or safety or tolerability reasons. The patient population is of particular interest due to evidence of decreased quality of life and increased economic burden among patients with migraine that is inadequately managed and is of interest to decision-makers globally.

**Methods:**

Key outcomes included overall mean change from baseline in monthly migraine headache days and proportions of patients achieving ≥30% (CM), ≥50%, and ≥ 75% reduction (response rates) in monthly migraine headache days across Months 1–3. Patient functioning and disability were evaluated at Month 3.

**Results:**

Of the 462 randomized patients, 186 (40.3%) had a history of 3–4 preventive category failures. Galcanezumab versus placebo resulted in significantly (*P* ≤ .001) larger overall mean reduction in monthly migraine headache days (total: − 5.49 versus − 1.03; CM: − 6.70 versus − 1.56; EM: − 3.64 versus − 0.65). Similarly, the ≥50% response rate was significantly (*P* ≤ .001) higher with galcanezumab versus placebo (total: 41.0 versus 12.7; CM: 41.5 versus 8.4; EM: 41.1 versus 16.5). In the CM group, the ≥30% response rate was significantly higher in the galcanezumab group than the placebo group (CM, 57.5 versus 19.8, *P* ≤ .0001) as was the ≥75% response rate (13.3 versus 2.6, *P* ≤ .05). Galcanezumab also resulted in significant (*P* < .0001) improvements in patient functioning and reductions in disability.

**Conclusions:**

Galcanezumab was effective in a difficult-to-treat population of patients with CM or EM who had failed 3–4 prior preventive medication categories.

**Trial registration:**

CONQUER. Clinicaltrials.gov identifier: NCT03559257.

## Background

Globally, migraine is second amongst the world’s causes of disability [1] and affects approximately one billion patients annually [2]. The prevalence of migraine peaks between the ages of 15 and 49 years. Despite the high prevalence, migraine remains under-treated [2–4]. Both chronic migraine (CM) and episodic migraine (EM) are associated with substantial headache-related disability, poor quality of life, and global societal burden [5, 6]. Evidence-based guidelines recommend the use of preventive medications for the management of patients with EM, with at least 4 headache days/month that are not adequately controlled by acute medications, and for patients suffering from CM. It is worth noting that when conventional oral preventive medications are prescribed, their limited effectiveness and high incidence of side effects lead to a high rate of discontinuation [7]. In particular, patients with CM who on an average switched between four preventive treatments had higher rates of discontinuations [8]. Patients with migraine who experienced failures with preventive treatment often rely on acute medications alone which may further aggravate the patient’s conditions, leading to disease progression, and associated higher disability, economic burden [8–11] and patients’ functioning [11–13]. The mean migraine-specific health care resource utilization (inpatient, outpatient, and emergency department visits) was higher in patients who switched to their fourth class of drug compared with patients who switched to either their third or those who never switched [14]. This resulted in mean total direct costs being significantly (*P* < .001) higher in patients who switched to their fourth ($5004) class of drugs within 1 year compared with patients who switched to either their third ($2997) or those who never switched ($2420) [14]. Newer, more effective, and better tolerated preventive treatment options are therefore required, particularly for patients with multiple preventive treatment failures.

Galcanezumab is a humanized monoclonal antibody that binds to calcitonin gene-related peptide and is approved for the prevention of migraine [15, 16]. In the EVOLVE and REGAIN studies, treatment with galcanezumab versus placebo resulted in a significant reduction in monthly migraine headache days [17–19]. Significant as well as clinically meaningful improvements in daily functioning and reduced disability were reported [20]. A subgroup analysis from the EVOLVE and REGAIN studies further demonstrated that galcanezumab compared with placebo resulted in significantly larger reduction in monthly migraine headache days, ≥50% response rates and functional improvement in patients with CM or EM with prior preventive treatment failure [21, 22]. However, these studies involved only a limited number of patients who had 3 or more medication category failures due to inadequate efficacy and were thus not designed to explore this patient population. However, this patient population, which qualified for the definition of treatment-resistant migraine ie, had failed ≥3 classes of migraine preventives and suffered from ≥8 debilitating headache days per month for ≥3 consecutive months without improvement [23] is of particular interest due to evidence of decreased quality of life and increased economic burden among patients with migraine that is inadequately managed [10, 14] and is of interest to decision-makers globally (eg, National Institute for Health and Care Excellence, Norwegian Medicines Agency, Pharmaceutical Benefits Advisory Committee) [24–27]. CONQUER (NCT03559257) was a phase 3, randomized, placebo-controlled study in patients with CM or EM and a history of failure to 2–4 prior preventive medications due to lack of efficacy and/or a safety/tolerability reason [28]. In this paper, we present the efficacy outcomes from a pre-specified analysis of galcanezumab 120 mg versus placebo in patients with 3–4 prior preventive medication category failures in the past 10 years due to inadequate efficacy (after at least 2 months at maximum tolerated dose), or safety or tolerability reasons.

## Methods

Data for this pre-specified analysis were drawn from the phase 3, multicenter, randomized, double-blind, placebo-controlled study in a subgroup of patients with CM or EM who had documented treatment failure of 3–4 standard-of-care migraine preventive medication categories in the past 10 years. A failure to a previous preventive medication was defined as inadequate efficacy (after at least 2 months at maximum tolerated dose), or safety or tolerability reasons. Contraindications did not count as medication failures. A detailed description of the study design has been published earlier [28]. Briefly, the CONQUER study consisted of an initial screening period, 1 month prospective baseline period, a three-month, double-blind treatment period, and an optional three-month open-label treatment period. During the three-month, double-blind treatment period, patients were randomized 1:1 to receive monthly subcutaneous injections of galcanezumab 120 mg (with a loading dose of 240 mg) or placebo.

Key inclusions were patients aged between 18 and 75 years, with a diagnosis of migraine as defined by International Classification of Headache Disorders version 3 (ICHD-3) guidelines [29] and treatment failure of 3–4 standard-of-care migraine preventive medication categories in the past 10 years due to inadequate efficacy (after at least 2 months at maximum tolerated dose), or safety or tolerability reasons. Patients who experienced more than four prior preventive medication category failures were excluded from the study. The preventive medication categories included propranolol or metoprolol, topiramate, valproate or divalproex, amitriptyline, flunarizine, candesartan and botulinum toxin A or B (if documented that botulinum toxin was taken for CM). Concomitant use of acute medications to treat migraine was allowed during the study with few limitations, such as restricted use of opioid- and barbiturate-containing medications (≤4 days/month) and injectable steroids (a single dose was allowed only once during the study in an emergency setting). Allowed concomitant acute headache treatment included acetaminophen, non-steroidal anti-inflammatory drugs, triptans, ergotamine and derivatives, dichloralphenazone and acetaminophen combination, or combinations thereof.

The study was conducted in concordance with the ethical principles that have their origin in the Declaration of Helsinki guidelines [30]. All patients provided written informed consent before study participation. The study protocol was reviewed and approved by the Institutional Review Board, Medical Ethics Committee, or Medical Research and Ethics Committee of the participating study sites.

### Study assessments

The outcomes were defined as per the primary manuscript [28] and include the overall mean change from baseline in monthly migraine headache days across Months 1 to 3 (where a migraine headache day was defined as a calendar day on which migraine headache or probable migraine headache occurs, that is a headache lasting ≥30 min, and having features meeting the criteria of the ICHD-3) [29]. Other outcomes assessed included changes in overall mean proportions of patients achieving ≥30% (for CM only), ≥50%, ≥75% and 100% reduction (response rates) in monthly migraine headache days assessed across Months 1 to 3. The mean change from baseline in the migraine-specific quality of life questionnaire (MSQ) version 2.1 total score, Role Function-Restrictive (RFR), Role Function-Preventive (RFP), Emotional Functioning (EF) domain score, and Migraine Disability Assessment (MIDAS) total scores were also assessed at Month 3.

### Statistical analysis

All analyses were performed on patients who were randomly assigned, received at least one dose of study drug and had failed at least 3 preventive medication categories. As per the primary manuscript [28], the overall mean change from baseline in monthly migraine headache days across Months 1–3 was determined using mixed-effects model repeated measures with effects for treatment, pooled country, month, treatment-by-month interaction, baseline, and baseline-by-month interaction. The response rates were calculated using categorical pseudo likelihood-based repeated measures model for binary outcomes with effects for treatment, month, treatment-by-month interaction, and baseline monthly migraine headache days; confidence limits were computed by applying inverse link transformation to the confidence limits on the logit scale, and could be asymmetric. The change from baseline in each of the three MSQ domain scores and the total score was analyzed with the same model as for the change from baseline in the monthly migraine headache days; however, the estimate at Month 3 was used instead of the overall estimate across Months 1 to 3.

Estimates for mean changes from baseline and response rates were obtained using unstructured covariance structures and the degrees of freedom were estimated using Kenward-Roger approximation. Two-sided *P* values ≤.050 were assumed to be statistically significant. No multiplicity adjustment strategy was implemented for these analyses. All statistical analyses were conducted using SAS software version 9.4 (SAS Institute Inc., Cary, NC, USA).

## Results

### Disposition and demographics

A total of 462 patients with CM or EM were randomized to receive galcanezumab 120 mg or placebo in the CONQUER study [28]. Among them, 40.3% (186/462) had a history of treatment failure of 3–4 standard-of-care migraine preventive medication categories according to the definition given in the Methods section and were included in this analysis. Table 1 summarizes the disposition and demographics among patients with 3–4 prior preventive medication category failures by migraine type.

**Table 1:** Baseline characteristics in patients with chronic migraine or episodic migraine who had failed 3–4 preventive medication categories due to inadequate efficacy, or safety or tolerability reasons within the last 10 years

	Total population		CM		EM	
	Placebo ***n*** = 87	GMB 120 mg ***n*** = 99	Placebo ***n*** = 43	GMB 120 mg ***n*** = 43	Placebo ***n*** = 44	GMB 120 mg ***n*** = 56
**Demographics**						
Age, years, mean (SD)	46.2 (13.2)	45.4 (10.7)	44.7 (14.1)	46.3 (10.7)	47.7 (12.3)	44.7 (10.7)
Gender (women), n (%)	77 (88.5)	78 (78.9)	37 (86.1)	35 (81.4)	40 (90.9)	43 (76.8)
Race, n (%)						
White	65 (75)	71 (72)	28 (65)	27 (63)	37 (84)	44 (79)
Asian	15 (17)	20 (20)	9 (21)	12 (28)	6 (14)	8 (14)
American Indian or Alaska Native	1 (1)	0 (0)	1 (2)	0 (0)	0 (0)	0 (0)
Not reported	6 (7)	8 (8)	5 (12)	4 (9)	1 (2)	4 (7)
BMI (kg/m^2^), mean (SD)	25.5 (6.0)	24.6 (5.0)	25.7 (5.9)	24.3 (5.3)	25.4 (6.1)	24.8 (4.8)
Total number of individual preventive medications that failed in the past 10 years, mean (SD)	4.6 (1.9)	4.5 (1.8)	5.3 (2.3)	4.9 (1.9)	3.9 (1.0)	4.2 (1.7)
Presence of aura at baseline, % (n/N)	46.0 (40/87)	42.4 (42/99)	46.5 (20/43)	48.8 (21/43)	45.5 (20/44)	37.5 (21/56)
**Disease characteristics**						
Time since initial migraine diagnosis, years, mean (SD)	23.4 (15.8)	22.4 (13.6)	24.2 (16.9)	24.8 (14.2)	22.6 (14.8)	20.5 (13.0)
Number of monthly migraine headache days, mean (SD)	14.1 (6.1)	14.0 (5.6)	18.8 (4.9)	18.8 (4.6)	9.5 (2.7)	10.2 (2.6)
Number of monthly migraine attacks, mean (SD)	6.0 (2.0)	6.0 (1.9)	6.3 (2.2)	6.0 (2.3)	5.7 (1.9)	6.0 (1.6)
Number of monthly headache days, mean (SD)	15.7 (6.1)	15.7 (6.2)	20.6 (4.4)	21.4 (4.7)	10.8 (2.4)	11.3 (2.5)
Number of monthly days with any acute headache medication use at baseline, mean (SD)	13.0 (6.1)	13.1 (5.9)	16.7 (6.0)	16.7 (6.9)	9.3 (3.4)	10.4 (3.0)
Migraine-Specific Quality of Life Questionnaire—Role Function-Restrictive domain score at baseline, mean (SD)	42.8 (19.6)	44.7 (17.4)	36.7 (18.3)	40.1 (19.5)	48.6 (19.3)	48.3 (14.9)
Migraine Disability Assessment total score at baseline, mean (SD)	57.5 (54.8)	52.0 (49.5)	81.2 (64.9)	66.5 (61.0)	34.4 (28.0)	41.0 (35.1)
Acute medication overuse, n (%)	39 (44.8)	60 (60.6)	30 (69.8)	33 (76.7)	9 (20.5)	27 (48.2)

*BMI* Body mass index, *CM* Chronic migraine, *EM* Episodic migraine, *GMB* Galcanezumab, *N* Number of patients in the analysis population with non-missing demographic measures, *n* number of patients within each specific category, *SD* Standard deviation

In the total population of patients with CM or EM who had failed 3–4 previous preventive treatments, the majority of patients were women (≥83%, 155/186) with a mean age of approximately 46 years and a mean duration of migraine since initial diagnosis of 23 years. The demographics and baseline characteristics for treatment groups are shown in Table 1.

No statistical difference between galcanezumab and placebo in the total population or in the subgroup of patients with CM or EM was observed at baseline in the variables presented in Table 1, except for medication overuse in the total population (*P* = .0393) and patients with EM (*P* = .0061), which was significantly higher in galcanezumab 120 mg compared with placebo. These two significant results at baseline are likely due to chance because of low sample sizes and post-hoc analyses performed on a factor not stratified within the randomization.

The patient disposition in this subgroup analysis was similar to the CONQUER primary study [28], except for certain differences such as a lower percentage of women (total, CM or EM), a higher total number of individual preventive medication failures over the past 10 years (total, CM or EM), a higher MIDAS total score and acute medication overuse in patients with CM.

### Reduction in monthly migraine headache days

**Fig. 1:**
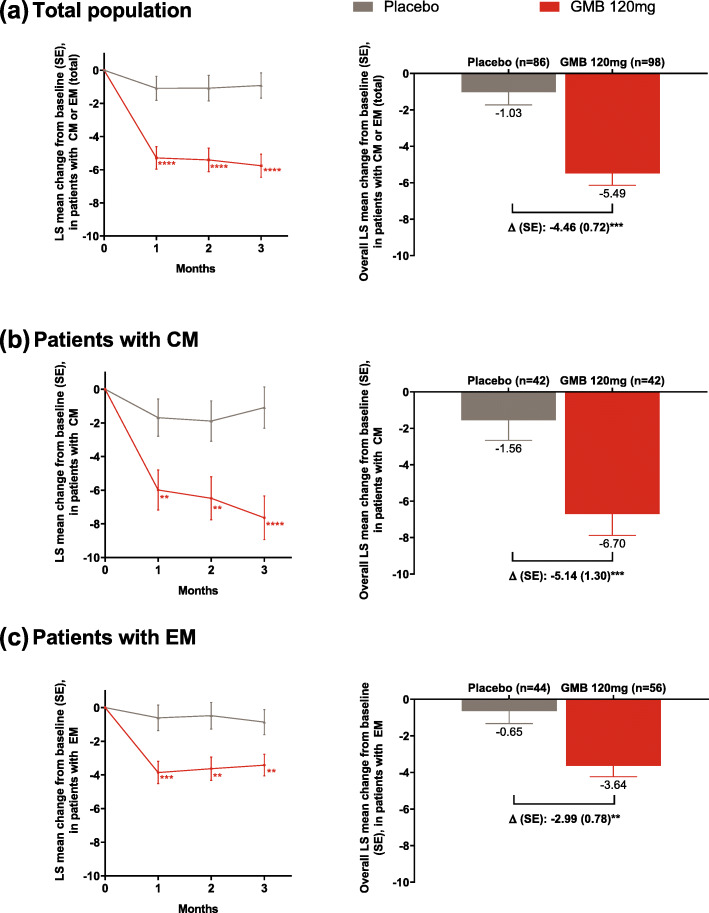
Monthly and overall least square (LS) mean changes from baseline in the number of migraine headache days during the treatment period in **a** the total population^#^, **b** patients with CM^#^ and **c** patients with EM^#^. ^#^Patients who had failed 3–4 preventive medication categories due to inadequate efficacy (after at least 2 months at maximum tolerated dose), or safety or tolerability reasons within the last 10 years. ****P ≤ .0001, ***P ≤ .001, **P ≤ .01 versus placebo. Abbreviations: CM, chronic migraine; EM, episodic migraine; GMB, galcanezumab; n, number of patients with non-missing baseline value and at least one post-baseline value; SE, standard error

In the total population, galcanezumab 120 mg versus placebo resulted in significantly larger overall mean reduction in monthly migraine headache days (− 5.49 versus − 1.03, respectively; Δ [standard error, SE], P: − 4.46 [0.72]; *P* ≤ .001; Fig. 1). Similar results were also observed in the subgroup of patients with CM (− 6.70 versus − 1.56; Δ [SE], P: − 5.14 [1.30]; *P* ≤ .001) and with EM (− 3.64 versus − 0.65; Δ [SE], P: − 2.99 [0.78]; *P* ≤ .001).

When considering monthly data, in the total population, galcanezumab 120 mg versus placebo led to a significantly larger reduction in migraine headache days at each month from Month 1 to Month 3. Similar results were also observed in the subgroup of patients with CM (Month 1 and 2, *P* ≤ .010; Month 3, *P* ≤ .0001) or EM (Month 1, *P* ≤ .0010, Month 2 and 3, *P* ≤ .010).

### Mean percentage of patients with ≥30%, ≥50% or ≥ 75% reduction from baseline in monthly migraine headache days

#### Total population

**Fig. 2:**
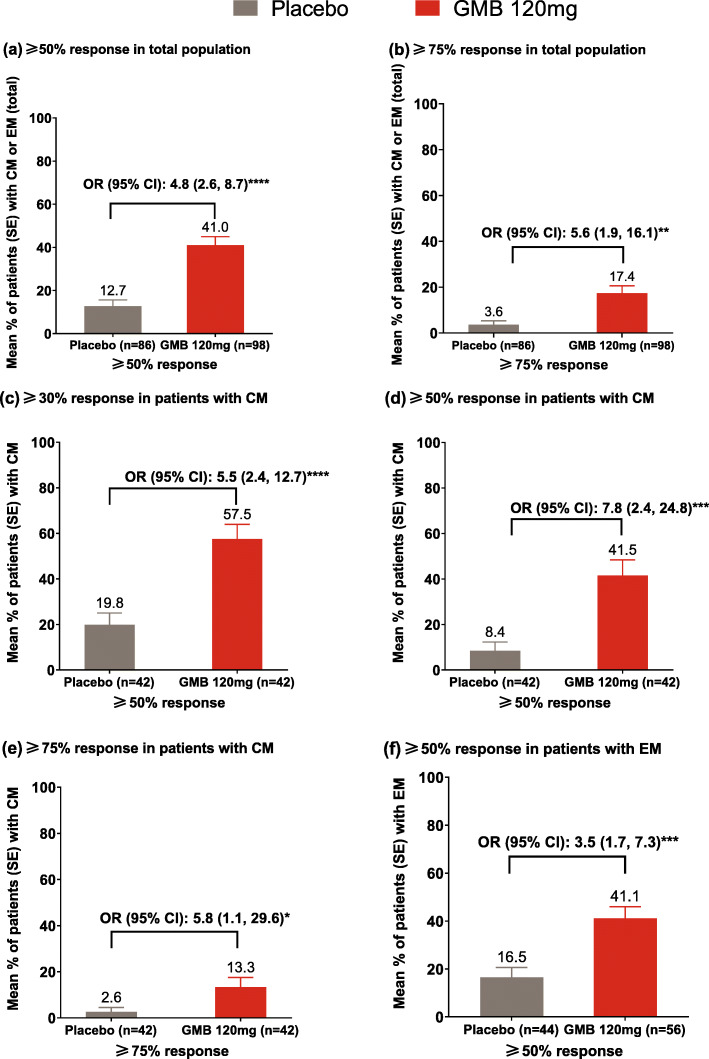
Mean percentage of patients with: **a** ≥50% and **b** ≥75% reduction in monthly migraine headache days (total population)^#^; **c** ≥30%, **d** ≥50%, and **e** ≥75% reduction in monthly migraine headache days in patients with CM^#^; and **f** ≥50% reduction in monthly migraine headache days in patients with EM^#^. Note: Only 30% response for patients with chronic migraine was pre-specified and was included in this analysis. ^#^ Patients who had failed 3–4 preventive medication categories due to inadequate efficacy (after at least 2 months at maximum tolerated dose), or safety or tolerability reasons within the last 10 years. ***P ≤ .001, **P ≤ 0.01, *P ≤ .05 versus placebo. CI: confidence interval; CM: chronic migraine; EM: episodic migraine; GMB: galcanezumab; LS: least squares; n: number of patients with non-missing baseline value and at least one post-baseline value; OR: odds ratio; SE: standard error

In the total population, the mean percentage of patients with ≥50% reduction from baseline in monthly migraine headache days was significantly (*P* ≤ .0001) greater with galcanezumab 120 mg versus placebo (41.0% versus 12.7%; odds ratio [OR] [95% confidence interval (CI)], P: 4.8 [2.6, 8.7]; *P* ≤ .0001, Fig. 2). The mean percentage of patients with ≥75% reduction from baseline in monthly migraine headache days was significantly larger with galcanezumab 120 mg versus placebo (17.4% versus 3.6%; OR [95% CI], P: 5.6 [1.9, 16.1]; *P* ≤ 0.010).

#### Chronic migraine subgroup

In the subgroup of patients with CM, galcanezumab 120 mg versus placebo resulted in significantly larger ≥30% reduction in monthly migraine headache days (57.5% versus 19.8%; OR [95% CI], P: 5.5 [2.4, 12.7]; *P* ≤ .0001). The ≥50% responders were significantly higher in the galcanezumab group compared with placebo (41.5 versus 8.4; OR [95% CI], P: 7.8 [2.4, 24.8]; *P* < .001). The same was also true for the ≥75% responders who were 13.3% in the galcanezumab group and 2.6% in the placebo group (OR [95% CI], P: 5.8 [1.1, 29.6]; *P* ≤ .050).

#### Episodic migraine subgroup

In the subgroup of patients with EM, the percentage of ≥50% responders was significantly higher in the galcanezumab group versus placebo (41.1 versus 16.5; OR [95% CI] P: 3.5 [1.7, 7.3]; *P* ≤ .001).

Due to the low sample size of patients with EM achieving ≥75% response in the placebo group (no patient at Month 1, two patients at Month 2 and four patients at Month 3), the overall rate could not be estimated with the planned analyses. Similarly, the 100% response rate could not be estimated in patients with CM or EM due to the low number of patients achieving this response rate.

### Patient functioning and disability

In the total population, galcanezumab 120 mg versus placebo resulted in significant mean improvements from baseline at Month 3 in the MSQ total score (26.0 versus 11.6; *P* < .0001), in the MSQ-RFR domain score (27.3 versus 12.0; *P* ≤ .0001), the MSQ-RFP domain score (21.4 versus 8.8; *P* < .0001), MSQ-EF domain scores (28.9 versus 14.5; *P* < .0001) and MIDAS total score (− 28.2 versus − 2.5; *P* < .0001; Table 2). Significant mean improvements with galcanezumab 120 mg versus placebo from baseline at Month 3 in MSQ total score, MSQ-RFR, MSQ-RFP and MSQ-EF domain scores were also observed in the subgroup of patients with CM or with EM (Table 2).

**Table 2:** Change from baseline in quality of life measures and number of monthly migraine headache days with acute medication use at Month 3^a^

Parameter	Total population		CM		EM	
	Placebo	GMB 120 mg	Placebo	GMB 120 mg	Placebo	GMB 120 mg
**MSQ total score**	*n* = 84	*n* = 94	*n* = 41	*n* = 40	n = 43	*n* = 54
Change from baseline, mean (SE)	11.6 (2.6)	26.0 (2.5)	5.7 (3.1)	24.4 (3.4)	13.6 (3.4)	22.1 (3.2)
Difference, mean (SE)	14.4 (2.8)		18.7 (3.9)		8.5 (3.8)	
P	<.0001		<.0001		0.0267	
**MSQ role function restrictive**	*n* = 84	*n* = 94	*n* = 41	*n* = 40	*n* = 43	*n* = 54
Change from baseline, mean (SE)	12.0 (2.8)	27.3 (2.7)	4.7 (3.4)	25.2 (3.6)	14.5 (3.6)	22.7 (3.4)
Difference, mean (SE)	15.3 (3.0)		20.5 (4.2)		8.2 (4.0)	
P	<.0001		<.0001		0.0426	
**MSQ role function emotional**	*n* = 84	*n* = 94	*n* = 41	*n* = 40	*n* = 43	*n* = 54
Change from baseline, mean (SE)	14.5 (3.2)	28.9 (3.1)	9.2 (4.0)	28.3 (4.4)	14.7 (4.1)	24.2 (4.0)
Difference, mean (SE)	14.4 (3.4)		19.0 (5.0)		9.5 (4.7)	
P	<.0001		0.0003		0.0479	
**MSQ role function preventive**	*n* = 84	*n* = 94	*n* = 41	*n* = 40	*n* = 43	*n* = 54
Change from baseline, mean (SE)	8.8 (2.5)	21.4 (2.4)	3.5 (3.1)	18.7 (3.3)	10.9 (3.2)	19.2 (3.0)
Difference, mean (SE)	12.6 (2.6)		15.2 (3.8)		8.3 (3.6)	
P	<.0001		.0001		.0233	
**Migraine Disability Assessment total score**	*n* = 85	*n* = 95	*n* = 42	*n* = 40	*n* = 43	*n* = 55
Change from baseline, mean (SE)	−2.5 (6.6)	−28.2 (6.5)	8.9 (10.5)	−31.0 (11.8)	−8.0 (5.4)	−18.2 (5.2)
Difference						
P	.0001		.0026		.084	
**Number of monthly migraine headache days with acute medication use**	*n* = 86	*n* = 98	n = 42	n = 42	n = 44	n = 56
Change from baseline, mean (SE)	−0.7 (0.7)	−5.3 (0.7)	−0.8 (1.0)	−7.0 (1.1)	−0.7 (0.8)	−3.5 (0.7)
Difference, mean (SE)	−4.6 (0.7)		−6.2 (1.8)		−2.8 (0.8)	
P	<.0001		<.0001		.0008	

*CM* Chronic migraine, *EM* Episodic migraine, *MSQ* Migraine-specific quality of life questionnaire, *SE* Standard error

^a^ Patients who had failed 3–4 preventive medication categories due to inadequate efficacy (after at least 2 months at maximum tolerated dose), or safety or tolerability reasons within the last 10 years

### Use of acute medications

Galcanezumab-treated patients in the total population had a significantly greater reduction in the number of monthly migraine headache days with acute medication use compared with placebo (− 5.3 versus − 0.7; mean change difference from placebo, − 4.6 (0.7); *P* < .0001). This result was similar in patients with CM or EM (CM: − 7.0 versus − 0.8; mean change difference from placebo, − 6.2 (1.8); *P* < .0001; EM: − 3.5 versus − 0.7; mean change difference from placebo, − 2.8 (0.8); *P =* .0008, Table 2).

**Fig. 3:**
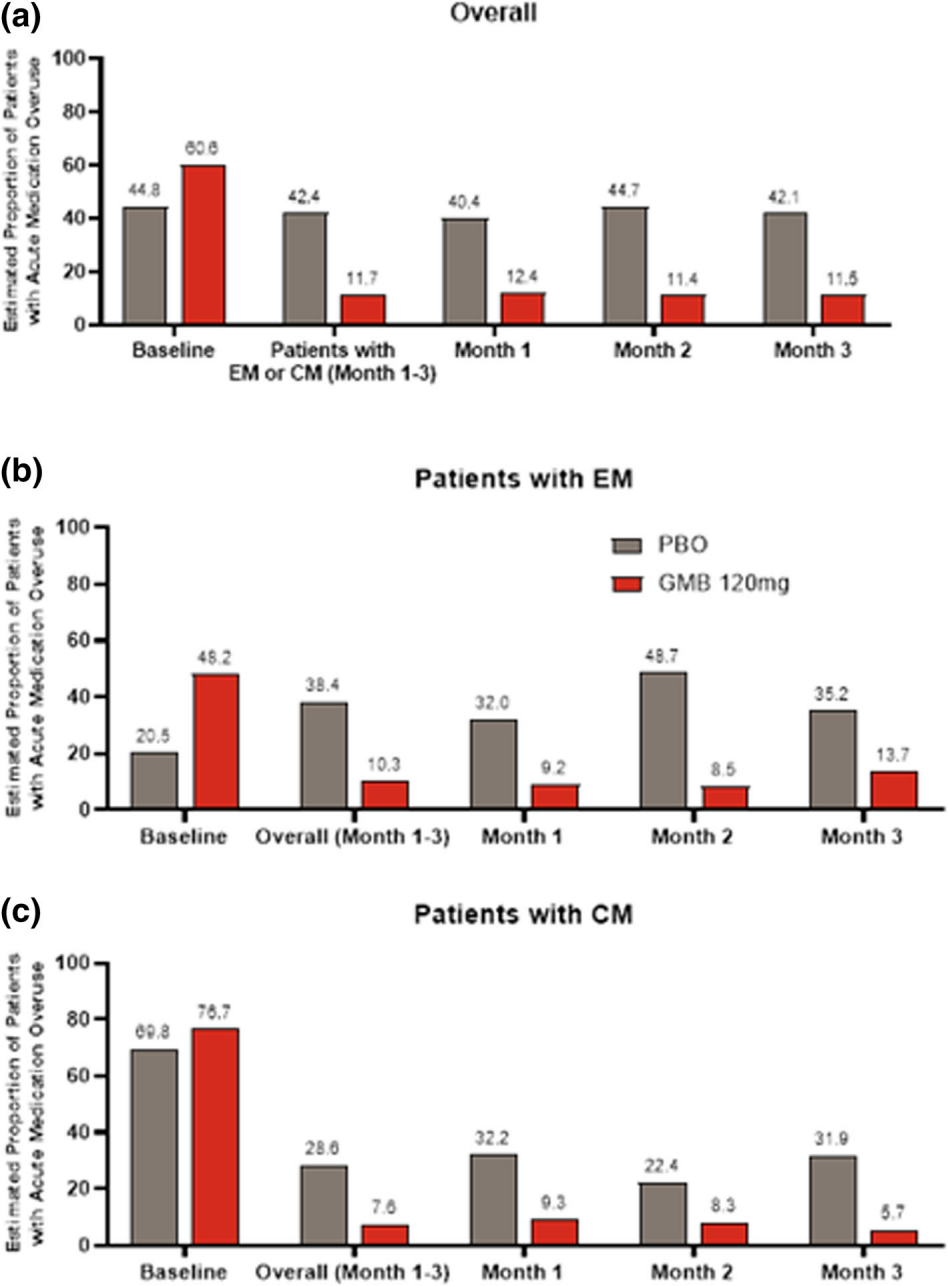
Estimated proportion of patients with acute medication overuse: **a**. Overall, **b** patients with episodic migraine and **c** patients with chronic migraine. Abbreviations: CM, chronic migraine; EM, episodic migraine; GMB, galcanezumab; PBO, placebo

Of note, galcanezumab 120 mg reduced the overall proportion of patients who satisfied the ICHD-3 criteria for acute medication overuse from 60.6% to 11.7 (Fig. 3).

## Discussion

Efficacy results from this pre-specified subgroup analysis of patients with CM or EM who had failed 3–4 preventive medication categories due to inadequate efficacy (after at least 2 months at maximum tolerated dose), or safety or tolerability reasons in the past 10 years demonstrate that galcanezumab 120 mg versus placebo is efficacious across multiple endpoints. In patients with CM, ≥30% reduction in monthly migraine headache days and in patients with EM, ≥50% reduction in monthly migraine headache days, is considered clinically meaningful [31–33]. In the present study, treatment with galcanezumab 120 mg versus placebo led to significantly larger ≥30% reductions in monthly migraine headache days in patients with CM and ≥ 50% reductions in monthly migraine headache days in patients with EM. Although direct comparisons are not warranted due to more severe patients in this subgroup analysis (3–4 versus 2–4 prior preventive medication category failures in CONQUER study), the efficacy parameters generally follow a similar pattern as observed in the intent-to-treat population of the CONQUER study [28].

Furthermore, the significantly larger mean reduction from baseline in monthly migraine headache days with galcanezumab 120 mg versus placebo observed in this subgroup analysis is consistent with earlier published subgroup findings [21, 22, 28]. The efficacy was accompanied by clinically significant improvement in functioning and a reduction in disability as shown by larger improvements in the MSQ-RFR domain and MIDAS total score with galcanezumab 120 mg versus placebo. Additionally, in this patient population, where acute medication use was high at baseline (Table 1), galcanezumab 120 mg versus placebo reduced significantly monthly days with any acute headache medications (Table 2) and markedly reduced the proportion of patients with acute medication overuse (Fig. 3a). This reduction in the use of acute medications is an indicator of the clinically significant effect of galcanezumab 120 mg versus placebo and is important in the management of patients with migraine who have a history of preventive treatment failures. Consistency with earlier published findings was also observed for other efficacy endpoints, including ≥50% reduction in monthly migraine headache and improvements in MSQ-RFR domain scores in patients with CM or EM with ≥2 prior preventive failures [21, 22, 28].

### Limitations

This is a pre-specified subgroup analysis of the larger population enrolled in the CONQUER study and, as such, included a smaller number of patients. These analyses were pre-specified, with the same models defined for the analyses on the full CONQUER population. Due to limited sample sizes and zero event count in the placebo group for the 75% responder definition, the planned model did not converge and therefore the difference between placebo and galcanezumab for the EM population could not be estimated. Analyses were not multiplicity adjusted.

## Conclusions

The current pre-specified analysis demonstrated the efficacy of galcanezumab versus placebo in reducing monthly migraine headache days in patients with CM or EM, who had documented treatment failure of 3–4 standard-of-care migraine preventive medication categories in the past 10 years owing to inadequate efficacy (after at least 2 months at maximum tolerated dose), or safety or tolerability reasons, or both. This population is of particular interest due to the evidence of decreased quality of life and increased economic burden among patients with migraine that is inadequately managed [10, 14] and is of interest to decision-makers globally (eg, National Institute for Health and Care Excellence, Norwegian Medicines Agency, Pharmaceutical Benefits Advisory Committee) [24–27]. More specifically, this analysis confirmed the efficacy of galcanezumab 120 mg versus placebo in ≥30% reduction in monthly migraine headache days in patients with CM and ≥ 50% reduction in monthly migraine headache days in patients with CM or EM, both of which are important thresholds for decision makers [24, 26, 34]. In addition, improvements in MSQ total score, as well as improvements in all MSQ domain scores, were also observed. Preventive medication is underused in the US and Europe [35, 36]. This is due to multiple factors including, but not limited to, lack of efficacy of particularly oral preventive treatments, poor tolerability, and suboptimal compliance. In this context, the efficacy of galcanezumab in the treatment of patients with treatment-resistant migraine has important implications for clinical practice, for the health system and, most of all, for patients who had previously experienced medication category failure to treatment interventions.

## Data Availability

Individual participant data collected during the trial, after anonymization, with the exception of pharmacokinetic or genetic data. Data are available to request 6 months after the indication studied has been approved in the US and EU and after primary publication acceptance, whichever is later. No expiration date of data requests is currently set once data are made available. Access is provided after a proposal has been approved by an independent review committee identified for this purpose and after receipt of a signed data sharing agreement. Data and documents, including the study protocol, statistical analysis plan, clinical study report, blank or annotated case report forms, will be provided in a secure data sharing environment. For details on submitting a request, see the instructions provided at www.vivli.org.
